# Chemical cross-linking to study protein self-assembly *in cellulo*

**DOI:** 10.1016/j.xpro.2024.103032

**Published:** 2024-04-22

**Authors:** Leonie Müller, Sirin Salman, Thorsten Hoppe

**Affiliations:** 1 Institute for Genetics, University of Cologne, 50674 Cologne, Germany; 2 Cologne Excellence Cluster on Cellular Stress Responses in Aging-Associated Diseases (CECAD), University of Cologne, 50931 Cologne, Germany; 3 Center for Molecular Medicine Cologne (CMMC), Faculty of Medicine and University Hospital of Cologne, 50931 Cologne, Germany

**Keywords:** Cell Biology, Molecular Biology, Protein Biochemistry

## Abstract

Many proteins self-assemble into dimers and higher-order oligomers. Therefore, the goal of this protocol is to characterize the conformational states of an endogenous protein of interest. Here, we present a protocol for assessing protein self-assembly in cell lysates using chemical cross-linking. We describe steps for chemical cross-linking with recombinant proteins as well as steps for cell culture and cell lysate preparation, chemical cross-linking, SDS-PAGE, and western blotting for the detection of endogenous proteins.

For complete details on the use and execution of this protocol, please refer to Balaji et al.^1^

## Highlights

• Instructions for cell culture and RNA transfection using HEK293T cells
• Unbiased chemical cross-linking to assess self-assembly
• Suitable for recombinant proteins and/or cell lysates

## Abstract (editor highlights)

Publisher’s note: Undertaking any experimental protocol requires adherence to local institutional guidelines for laboratory safety and ethics.

## Graphical abstract

**Figure:**
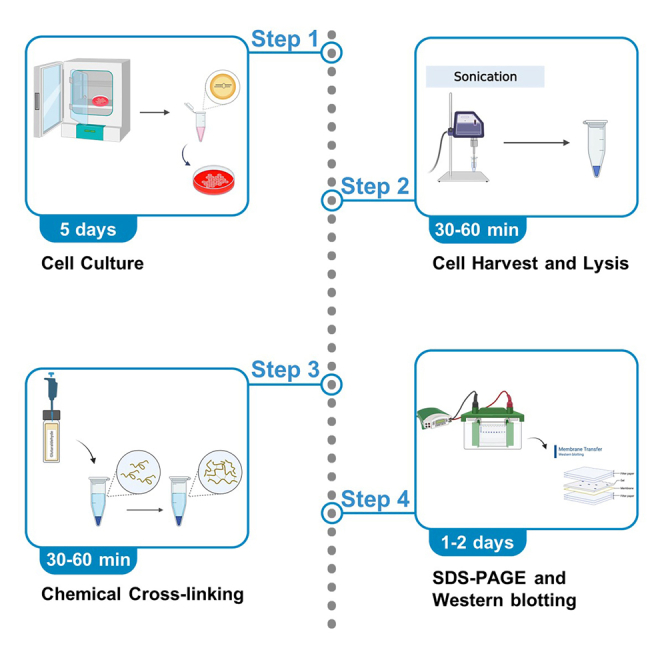

## Before you begin

Protein self-assembly plays a central role in many biophysical and biological processes. Several theories have emerged to explain why nature favors this phenomenon, as discussed in various reviews.^2^^,^^3^^,^^4^^,^^5^ While some proteins dimerize in response to specific cellular stimuli, others exist predominantly as dimeric or oligomeric structures. In the latter case, it is generally assumed that self-assembly occurs rapidly after de novo folding, resulting in low levels of free monomers in the cell.

Therefore, the primary challenge is to detect and analyze endogenous monomeric structures. However, very little research has been done to elucidate whether the monomeric structure of these functional, self-assembled structures themselves serve a different function than their dimeric and oligomeric counterparts. Deciphering the conformational state of proteins is essential for assessing the various functions and pathways in which the protein may be involved in. To the best of our knowledge, we provide the first protocol for the analysis of conformational changes in endogenous proteins in cell lysates.

The provided protocol is designed to analyze protein self-assembly *in ex vivo* using cell lysates. Specifically, we describe the protocol for using HEK293T cells, which endogenously express our protein of interest, the C-terminal HSP70-interacting protein (CHIP). Alternatively, other cell lines may be used. By using HEK293T cells in combination with either small interfering RNA (siRNA) to knock down endogenous CHIP^6^ or non-targeting small RNA (scRNA) as a control,^6^ the conformational states of CHIP after cross-linking can be analyzed by Western blotting. We have recently used the same cross-linking protocol for mouse embryonic fibroblasts and HeLa cells, for CHIP overexpression studies, and for recombinant CHIP proteins.^1^

For cross-linking with recombinant proteins, prepare and store all required buffers and solutions, including HEPES Buffer, Tris Buffer, 0.5% Glutaraldehyde Crosslinking Solution, 4× LDS Sample Buffer, 1× MES Running Buffer, and Coomassie Stain (see materials and equipment and key resources table). Except for the Coomassie Stain, these buffers and solutions will be used for the crosslinking protocol with cell lysates as well. The recombinant protein can be from any organism and is stored in storage buffer (50 mM Tris-HCl pH 7.5, 150 mM NaCl, 5 mM DTT, 5% glycerol). For crosslinking with cell lysates, prepare and store all required buffers and solutions (see materials and equipment and key resources table). Maintain HEK293T cells at 37°C under 5% CO_2_ in GlutaMAX medium (see key resources table), supplemented with 10% fetal bovine serum (FBS) and 1% penicillin/streptomycin (PS).

### Chemical cross-linking using recombinant proteins

**Timing: 30–60 min**

The purpose of this step is to evaluate the conditions such as cross-linking time points and glutaraldehyde concentration needed to assess the conformational state of your protein of interest.

Glutaraldehyde is a homobifunctional cross-linking reagent with two reactive carbonyl groups at each end that interact with primary amines (NH_2_-R). Primary amines are found at the N-terminus of every protein. Because glutaraldehyde requires a certain minimum distance for primary amines to react, only proteins in close proximity will be covalently cross-linked. Therefore, cross-linked proteins are considered to be interacting proteins.

1. Incubate 3 μM of the recombinant protein of interest in HEPES buffer to a final volume of 20 μL for 10 min at 30°C using a heating block.
  **CRITICAL:** The recombinant protein should have a concentration of approximately 1 μg/μL. This ensures that the volume of HEPES buffer added to the recombinant protein effectively dilutes the storage buffer components that could interfere with the crosslinking reaction (see troubleshooting).
  a. Calculate the amount of recombinant protein needed to obtain 3 μM in 20 μL. Add this volume to a fresh 1 mL Eppendorf tube and make up to 20 μL with HEPES buffer.
  b. To obtain the protein concentration in μM, divide the concentration of your protein in ng/μL by the molecular mass of your protein of interest in kDa.
    ***Note:*** Prepare two reactions for each protein of interest to obtain a sample for the cross-linking reaction and a control sample.
2. After 10 min at 30°C, add 1 μL of 0.5% glutaraldehyde to your cross-linking sample but not to your control sample. The final concentration of glutaraldehyde per reaction is 0.025%.
3. Incubate both samples for an additional 10 min at 30°C.
4. Quench the cross-linking reaction by adding 2 μL of 1 M Tris buffer. Also add Tris buffer to the control sample. The final Tris buffer concentration is 95 mM.
5. Incubate all samples at 20°C–22°C for 15 min.
6. Add 7.5 μL of 4× LDS sample (without reducing agent) to each sample and incubate for 10 min at 70°C.

**Pause point:** After LDS buffer treatment, samples can be stored at −20°C and SDS-PAGE can be performed the next day.

7. Perform SDS-PAGE as described in “step-by-step method details”. As a final step, place your gel in a plastic container filled with Coomassie Stain (instead of semi-dry blot buffer).
8. Incubate the gel for 12–18 h at 20°C–22°C.
9. The next day, wash the gel by exchanging the Coomassie Stain with water and incubate until a clear background contrasts with the blue protein bands.

***Note:*** The use of a protein tag fused to the recombinant protein of interest is not recommended because the tag may interfere with the crosslinking reaction. The effect of the tag depends on the properties of both the protein tag and the protein of interest. To generate an untagged protein, choose a protein tag that can be cleaved after purification. If you cannot avoid using a protein tag, use a small protein tag without primary amines, such as the hexahistidine (His) tag.

***Alternatives:*** In addition to the suggested storage buffer, recombinant proteins can be stored in other buffers prior to the crosslinking experiment. However, depending on the crosslinking agent, some issues should be considered that are discussed in the “troubleshooting” section.

The result of the Coomassie-stained gel for our protein of interest, CHIP, is shown in the “Expected results” section. In addition to wild-type CHIP, we used a mutant version of CHIP that contains a point mutation in its dimerization interphase (L165R), resulting in a monomeric form of CHIP that cannot self-assemble. As expected, CHIP(L165R) remains predominantly monomeric after 10 min of cross-linking. Some dimerization of CHIP(L165R) is observed, resulting from concentration-dependent oligomerization.

## Key resources table

**Table:** 

REAGENT or RESOURCE	SOURCE	IDENTIFIER
**Antibodies**		

Rabbit monoclonal anti-CHIP (C3B6)	Cell Signaling Technology	Cat#2080, RRID: AB_2198052
Peroxidase-conjugated AffiniPure mouse anti-rabbit IgG+IgM	Jackson ImmunoResearch	Cat# 111-035-144, RRID: AB_2307391

**Chemicals, peptides, and recombinant proteins**		

Glutaraldehyde solution	Sigma-Aldrich	Cat#49629-205ML
RotiBlock	Carl Roth	Cat#A151.1
Prestained protein ladder (PageRuler Plus)	Thermo Fisher Scientific	Cat#11852124
Quick Coomassie stain	Protein Ark	Cat#GEN-QC-STAIN-1L
NaCl	Roth	Cat#P029.2
KCl	Roth	Cat#6781.1
MES	Roth	Cat#4256.4
EDTA	Sigma-Aldrich	Cat#E5134
HEPES	Sigma-Aldrich	Cat#54457-250G-F
Tris-HCl	Roth	Cat#9090.3
Na_2_HPO_4_	Roth	Cat#P303.3
KH_2_PO_4_	Roth	Cat#P018.3
Methanol	VWR	Cat#20847.307
Triton X-100	Thermo Fisher Scientific	Cat#HFH10
Tween 20	Roth	Cat#9127.2
Pefabloc SC Plus	Roche	11873601001
c Omplete Tablets EDTA-free Easy Pack (Protease Inhibitor Cocktail Tablets)	Roche	4693132001
ROTI Stock 20% SDS	Roth	Cat#1057.1

**Critical commercial assays**		

ECL Prime western blotting detection reagents	Cytiva Amersham	Cat#GERPN3243
Lipofectamine RNAi MAX	Thermo Fisher Scientific	Cat#13778075

**Experimental models: Cell lines**		

HEK293T (human embryonic kidney cells)	Sigma-Aldrich	Cat#85120602

**Oligonucleotides**		

siRNA targeting human CHIP: Hs_STUB1_1	QIAGEN	GeneGlobe ID-SI00081963
siSCR: sense 5-(GGA UUA CUU GAU AAC GCU AUU) TT-30 Antisense 5-(AAU AGC GUU AUC AAG UAA UCC) TT-30	Albert et al.^7^	N/A

**Software and algorithms**		

Image Lab 6.1.0 build 7	Bio-Rad Laboratories, Inc.	www.bio-rad.com/de-de/product/image-lab-software?ID = KRE6P5E8Z
BioRender	BioRender	www.biorender.com
Inkscape - draw freely	Inkscape	www.inkscape.org

**Other**		

Amersham Protran 0.1 μm NC	GE Healthcare	Cat#10600000
4× LDS sample buffer	Novex	Cat#B0007
NuPAGE antioxidant	Novex	Cat#NP0005
NuPAGE transfer buffer (20×)	Novex	Cat#NP0006-1
DMEM, high glucose, GlutaMAX supplement	Thermo Fisher Scientific	Cat#10566016
Opti-MEM	Gibco	Cat#31985070
NuPAGE 10%, Bis-Tris	Thermo Fisher Scientific	Cat#NP0301BOX
Extra thick blot paper	Bio-Rad	Cat#1703966

## Materials and equipment

***Alternatives:*** BioRender (see key resources table) is a commercially purchased software used to create the graphical abstract. However, the use of BioRender can be replaced by the free software Inkscape.

Buffers and reagents prepared manually in the laboratory are listed below. All other buffers and reagents used in this protocol were purchased from various sources and used according to the manufacturer’s instructions. All buffers and solutions can be stored at 20°C–22°C and reused unless otherwise stated.

***Alternatives:*** If available, all reagents and equipment used may be purchased from other companies.

**Table:** 10× Phosphate-Buffered Saline (10× PBS)

Reagent	Final concentration	Amount
NaCl	1.37 M	80.06 g
KCl	27 mM	2.01 g
Na_2_HPO_4_ 2H_2_O	80 mM	14.24 g
KH_2_PO_4_	20 mM	2.72 g
ddH_2_O	N/A	∼900 mL
**Total**	**N/A**	**1000 mL**

***Note:*** Autoclave for 15 min at 121°C and 98.9 kPa. For 1× PBS, dilute stock solution with ddH_2_O. Store at 20°C–22°C for a maximum shelf life of 2 years (or until the earliest expiration date of one of the reagents).

**Table:** Native Lysis Buffer

Reagent	Final concentration	Amount
HEPES	50 mM	5.96 g
NaCl	150 mM	4.38 g
EDTA	1 mM	0.19 g
Triton X-100	1%	5 mL
ddH_2_O	N/A	∼500 mL
**Total**	**N/A**	**500 mL**

**CRITICAL:** Immediately before use, add 1× Protease Inhibitor Cocktail (Roche) and 1 mM Pefabloc to the lysis buffer. Pefabloc is a non-toxic alternative to PMSF (protease inhibitor), stable in aqueous solution. After addition, the lysis buffer should be used up completely. If you would like to reuse the lysis buffer later, remove your required volume (according to the number of samples you have) and add the 1× Protease Inhibitor Cocktail (Roche) and 1 mM Pefabloc to the volume of Lysis buffer needed. Store the remaining volume of Lysis buffer at 4°C (without 1× Protease Inhibitor Cocktail (Roche) and 1 mM Pefabloc). Be sure to only add the 1× Protease Inhibitor Cocktail (Roche) and 1 mM Pefabloc to the lysis buffer immediately before use.

***Note:*** Adjust pH to 7.4 with hydrochloric acid. Store at 4°C for a maximum shelf life of 2 years (or until the earliest expiration date of one of the reagents).

***Alternatives:*** Alternative lysis buffers can be used. However, they should be “native lysis buffers” characterized by preservation of the native conformations of proteins. This is typically achieved by using physiological pH and salt concentrations, mild detergents, good buffering capacity, and no denaturants.

**Table:** HEPES Buffer

Reagent	Final concentration	Amount
HEPES	50 mM	2.38 g
NaCl	150 mM	1.75 g
ddH_2_O	N/A	∼ 200 mL
**Total**	**N/A**	**200 mL**

**CRITICAL:** Allow the buffer to reach 20°C–22°C before use for optimal cross-linking efficiency.

***Note:*** Store at 20°C–22°C or 4°C for a maximum shelf life of 2 years (or until the earliest expiration date of one of the reagents).

**Table:** 1× MES Running Buffer

Reagent	Final concentration	Amount
MES	50 mM	9.76 g
Tris base	50 mM	6.06 g
SDS	3.47 mM	1 g
EDTA	1.03 mM	0.3 g
ddH_2_O	N/A	∼1000 mL
**Total**	**N/A**	**1000 mL**

***Note:*** Following the exact recipe above, the pH of the MES buffer is 7.6. Do not adjust pH with acid or base since MES itself is a buffer component. Autoclave for 15 min at 121°C and 98.9 kPa. Store at 20°C–22°C or at 4°C for a maximum shelf life of 2 years (or until the earliest expiration date of one of the reagents).

***Alternatives:*** Alternative SDS-PAGE buffers are available. The choice of buffer depends on the gel being used. For Bis-Tris gels, MES or MOPS running buffers are commonly used. The choice between MES and MOPS also depends on the molecular mass of the protein of interest, as each buffer may result in different size resolution and running behavior.

**Table:** Semi-dry Blot Buffer

Reagent	Final concentration	Amount
NuPAGE Antioxidant Reagent (see key resources table)	N/A	0.5 mL
20× NuPAGE transfer buffer (see key resources table)	2×	50 mL
MeOH	100%	50 mL
ddH_2_O	N/A	399.5 mL
**Total**	**N/A**	**500 mL**

***Note:*** Store at 20°C–22°C or at 4°C for a maximum shelf life of 6 months (or until the earliest expiration date of one of the reagents). Since MeOH is highly volatile, close the buffer properly.

**CRITICAL:** MeOH is toxic and can form extremely high vapor concentrations at room temperature. Always use it within a ventilated fume hood and away from any source of ignition as it is flammable.

**Table:** Primary Antibody

Reagent	Final concentration	Amount
PBS-T	1× PBS, 0.1% Tween	∼9 mL
RotiBlock	1×	1 mL
Primary Antibody	1:5000	2 μL
**Total**	**N/A**	**10 mL**

***Note:*** Thaw primary antibody stock solution on ice. Keep at 4°C prior to use. Store the antibody stock at −20°C or −80°C. The primary antibody solution may be reused once or twice. For reuse, store the antibody solution at 4°C or −20/−80°C.

**Table:** Secondary Antibody

Reagent	Final concentration	Amount
PBS-T	1× PBS, 0.1% Tween	∼9 mL
RotiBlock	1×	1 mL
Primary Antibody	1:10000	1 μL
**Total**	**N/A**	**10 mL**

***Note:*** Thaw secondary antibody stock solution on ice. Keep at 4°C prior to use. Store the antibody stock at −20°C or −80°C. We suggest to not reuse the secondary antibody solution.

• **0.5% Glutaraldehyde Cross-linking Solution:** Dilute 0.1 mL of the 50% glutaraldehyde stock solution in 4.9 mL ddH_2_O to obtain a 1% solution. Then add another 5 mL ddH_2_O to obtain a final concentration of 0.5% glutaraldehyde.

**CRITICAL:** Glutaraldehyde is toxic and affects the respiratory system. Side effects may include skin irritation. Be aware of the hazard before use. Store it at 20°C–22°C and protect from light. Wear protective gloves and goggles when handling and pipette the glutaraldehyde solution under a fume hood.

**CRITICAL:** Always prepare fresh before use and protect from light (e.g. with aluminum foil).

***Alternatives:*** The use of other cross-linking agents is possible. However, this protocol is specifically designed to use Glutaraldehyde. Although there are alternative cross-linking agents that also act on primary amines, such as Formaldehyde, Disuccinimidyl suberate (DSS), and Bis(sulfosuccinimidyl) suberate (BS3), we cannot guarantee that this protocol will work equally well with them.

• **TRIS Buffer:** The amount of buffer depends on the number of cross-linking reactions. Prepare a final concentration of 1 M Tris-HCl in ddH_2_O. Adjust the pH to 7.5 with hydrochloric acid.

***Note:*** Store at 20°C–22°C or 4°C for a maximum shelf life of 2 years (or until the earliest expiration date of one of the reagents).

• **PBS-T:** Prepare PBS-Tween (PBS-T) by adding 1 mL 100% Tween to 1 L of 1× PBS (final 0.1% Tween in 1× PBS).

***Note:*** Store at 20°C–22°C or 4°C for a maximum shelf life of 2 years (or until the earliest expiration date of one of the reagents).

• **Blocking Solution:** Prepare 200 mL of blocking solution (5% milk) by dissolving 10 g milk powder in 200 mL 1× PBS-T.

***Note:*** Store at 4°C for up to one week.

Equipment for cell culture.

• TC Dish 100, Standard (Petri dishes (Ø 10 cm)).
• Microcentrifuge 5425 with rotor FA-45-24-11.
• Centrifuge 5810 R with rotor A-4-62.
• Cell Scraper 28 cm length.
• Leica DM IL LED.
• Neubauer chamber.

## Step-by-step method details

### Cell culture and RNA transfection using HEK293T cells

**Timing: 5 days**

This section describes the steps for HEK293T cell preparation and RNAi transfection.

1. Seed cells in Petri dishes (Ø 10 cm).
  a. At 40–60% HEK293T cell confluence, seed 2e^6^ cells per petri dish.
    i. Count cells using a Neubauer chamber and calculate the volume needed for 2e^6^ cells.
    ii. Add the required volume to the Petri dish and make up to 10 mL with pre-warmed medium.
    iii. Use 2e^6^ of cells/petri dish/condition.
      ***Note:*** Here, we need two Petri dishes for siRNA and scRNA treatment, respectively.
  b. Incubate the cells for 24 h at 37°C/5% CO_2_. After 24 h, the cells should have reached approximately 60–80% confluence.
2. Transfect cells with siRNA (CHIP knockdown) or scRNA (control).
  a. Prepare the RNA-lipid complexes required for transfection.
    i. Dilute 18 μL Lipofectamine RNAiMAX Reagent per condition in 300 μL Opti-MEM medium.
    ii. Dilute 6 μL of 10 μM siRNA and 6 μL of 10 μM control RNA (final amount of RNA is 30 pmol) each in 300 μL Opti-MEM medium.
    iii. Combine the diluted Lipofectamine RNAiMAX Reagent with the diluted siRNA or scRNA (1:1 ratio) and incubate for 5 min at 20°C–22°C to allow RNA-lipid complexes to form.
  b. Add the entire volume of the prepared siRNA-lipid complex or scRNA-lipid complex from the previous step dropwise to the cells.
  c. Incubate the cells for 72 h at 37°C/5% CO_2_.

### HEK293T cell lysate preparation

**Timing: 30–60 min**

The purpose of this step is the extraction of cellular components, such as proteins, required for downstream analysis. It yields a homogenized sample suitable for biochemical analysis.

3. Perform cell lysis by sonication.
  a. Use a cell scraper/conditioner to gently scrape adherent cells from the surface of the culture flask and collect each cell suspension in a 15 mL Falcon tube.
  b. Wash the cells twice with ice-cold PBS.
    i. Spin the cells at 1500 rpm/435 × *g* for 5 min at 4°C using a Centrifuge (5810 R with rotor A-4-62).
    ii. Carefully remove the medium without touching the cell pellet and resuspend the cell pellet in 10 mL PBS.
    iii. Repeat the centrifugation step, remove the PBS, and wash again.
  c. After the last wash, remove as much PBS as possible without touching the cell pellet.
  d. Add 150 μL ice-cold native lysis buffer to each cell pellet and gently resuspend by pipetting up and down.
    ***Note:*** The pellet may not be completely dissolved at this time.
    i. Cut the 1000 μL pipette tip at approximately 2 mm to avoid the pellet getting stuck in the tip.
    ii. Carefully transfer the cell suspension to a fresh 1 mL Eppendorf tube, taking care not to lose any of the pellet.
  e. Lyse the cells by sonication using the Bioruptor Pico (Diagenode) at 60% amplitude with 10 cycles of 30 s ON and 30 s OFF at 4°C.
    ***Note:*** The pellet should be completely resuspended at the end of this step.
    i. Centrifuge the lysed cells at 15,000 × *g* (Microcentrifuge 5425 with rotor FA-45-24-11) for 20 min at 4°C to separate the pelleted cell debris from the clear lysate.
  f. Carefully transfer the supernatant-containing cell lysate to a 1 mL Eppendorf tube and store it on ice.
  g. Select a method of your choice to quantify the protein concentration of the lysate.
    ***Note:*** We typically accomplish this by subjecting a defined volume of sample (e.g. 10 μL) to SDS-PAGE analysis and Coomassie staining in conjunction with a dilution series of BSA samples with known protein concentrations. After quantification of the band intensities, the concentration of the lysate sample can be calculated from the BSA standard curve.
    **CRITICAL:** The most promising approach is to proceed immediately with the cross-linking procedure. If you must freeze the lysates, freeze them only once in liquid nitrogen and use them immediately the next time. Freezing and thawing adversely affects the efficiency of cross-linking and thus the quality of the data.

### Chemical cross-linking using HEK293T cell lysates

**Timing: 30–60 min**

The purpose of this cross-linking step is to preserve interactions between different protein subunits, protein domains, and other proteins that are in close proximity to each other. Since glutaraldehyde requires a certain minimum distance for primary amines to react, only proteins in close proximity will be covalently cross-linked.

4. Incubate 80 μg of total cell lysate in HEPES buffer to a final volume of 20 μL for 10 min at 30°C using a heating block.
  a. Calculate the amount of cell lysate required to obtain 80 μg of total protein. Transfer this volume to a fresh 1 mL Eppendorf tube and make up to 20 μL with HEPES buffer.
5. After 10 min at 30°C, add 1 μL of 0.5% glutaraldehyde to your cross-linking samples, but not to your control samples.

***Note:*** The final concentration of glutaraldehyde per reaction is 0.025%.

6. Incubate all samples for 1 min at 30°C.
  a. Incubate samples with glutaraldehyde according to your optimized cross-linking time points (See “troubleshooting”).
7. Quench the cross-linking reaction by adding 2 μL of 1 M TRIS buffer. Add TRIS buffer to the control samples as well.

***Note:*** The final concentration of the TRIS buffer per reaction is 95 mM.

8. Incubate all samples for 15 min at 20°C–22°C.
9. Add 7.5 μL of 4× LDS buffer (without reducing agent) to each sample and incubate for 10 min at 70°C.

**Pause point:** After LDS buffer treatment, samples can be stored at −20°C and SDS-PAGE can be performed the next day.

10. Perform SDS-PAGE and Western blot analysis.

**CRITICAL:** The incubation time with 0.5% glutaraldehyde is critical to the success of your experiment. If you exceed an incubation time of 10 min, all CHIP dimers will be cross-linked to high order oligomers. The time to stop the cross-linking reaction after 1 min has been optimized for CHIP. For a novel protein of interest, it is highly recommended to optimize the end point of the cross-linking reaction (see troubleshooting). For SDS-PAGE sample preparation, do not exceed 10 min at 70°C. Boiling of samples will break the covalent bonds of cross-linked proteins.

### SDS-PAGE

**Timing: 1 h**

The purpose of this step is to separate proteins obtained from the cell lysates (or recombinant proteins) based on their molecular weight for further downstream analysis.

11. Load the SDS-PAGE gel.
  a. Load the SDS-PAGE chamber with a 10% Bis-Tris gel and fill the chamber with MES running buffer.
  b. To clean the wells of the gel, pipette MES buffer in and out of the wells.
  c. Load 5 μL protein ladder and 20 μL protein samples.
12. Run the gel at 160 V for approximately 40 min so that the sample front reaches the bottom of the gel.

***Note:*** The running conditions are optimized for CHIP, which has a molecular weight of 35 kDa. The settings described result in a broad size separation on the membrane, between approximately 10 kDa at the bottom and 190 kDa at the top of the membrane, which is suitable for separating CHIP monomers, dimers, and oligomers. For significantly larger or smaller proteins, the settings may need to be optimized. For example, if your monomeric protein is already 70 kDa, you can run the sample front until the 50 kDa band of the protein ladder reaches the bottom of the gel.

13. Carefully break the plastic cast of the gel by inserting a spatula into the bottom of the plastic cast.
14. Cut off the stacking portion of the gel and gently remove the gel. Moisten your gloves to avoid breaking the gel.
15. Transfer the gel to a plastic container filled with semi-dry blotting buffer and proceed with Western blotting.

### Western blotting

**Timing: 1–2 days**

This step provides information necessary for assessing the conformational states of the protein of interest. Based on the intensity of the bands that appear at different molecular weights in the Western blot, the conformational state (i.e., monomer, dimer, oligomer) of your protein of interest can be assessed.

16. Cut a gel-sized piece of nitrocellulose membrane and incubate in a plastic container filled with semi-dry blotting buffer for 1–5 min at 20°C–22°C.
17. Cut two gel-sized pieces of blotting paper and incubate in a plastic container filled with semi-dry blotting buffer for 1–5 min at 20°C–22°C.
18. Prepare the Western blot (WB) sandwich.
  a. Assemble the WB sandwich in a WB chamber from the bottom up in the following order: Blotting paper, nitrocellulose membrane, SDS gel, blotting paper.
  b. Gently roll the sandwich several times to remove any air bubbles that may be trapped between the sandwich layers.
  c. Close the chamber and allow excess blotting buffer to drain from the chamber.
19. Perform a semi-dry transfer.
  a. Place the chamber in the appropriate blotting device and use the following semi-dry blotting program: 25 V, 1.0 A, 30 min.
  b. Transfer the blotted membrane to a plastic container filled with blocking solution and incubate for 30 min at 20°C–22°C.
20. Perform antibody staining.
  a. Incubate the membrane with an appropriate dilution of primary antibody (here 1:5000 for rabbit monoclonal anti-CHIP) in PBS-T/1× RotiBlock (e.g., 9 mL PBS-T and 1 mL 10× RotiBlock). Incubate for 1 h at 20°C–22°C or 16–18 h at 4°C. RotiBlock is used as the blocking reagent.
  b. Wash the membrane 3 times with PBS-T for 10 min at 20°C–22°C.
  c. Incubate the membrane with an appropriate dilution of secondary antibody in PBST/RotiBlock (here 1:10000 for mouse anti-rabbit) for 1 h at 20°C–22°C.
  d. Wash the membrane 3 times with PBS-T for 5 min at 20°C–22°C.
21. Develop the Western blot.
  a. Immediately before use, mix the two ECL Prime Western Blotting Detection Reagents (see key resources table) in a 1:1 ratio (e.g., using 1 mL each) and transfer the detection solution onto the membrane.
    ***Note:*** Here, we used a horseradish peroxidase (HRP)-conjugated secondary antibody with ECL Prime Western Blotting Detection Reagents.
  b. Capture the HRP signal using a charge-coupled device camera. The exposure time depends on the protein of interest.
    ***Note:*** For CHIP, we used a charge-coupled device using the fast auto exposure option in the device (exposure time of 24.63 s).
    ***Alternatives:*** The use of an HRP-conjugated secondary antibody is not mandatory and may be substituted at the user’s discretion. However, the detection method should be very sensitive because endogenous monomers may be present in the cell at very low levels compared to dimeric and oligomeric counterparts. We use HRP-conjugated secondary antibodies in combination with ECL Prime Western Blotting Detection Reagents (see key resources table) to obtain the best Western blot signal.

## Expected outcomes

**Figure 1:**
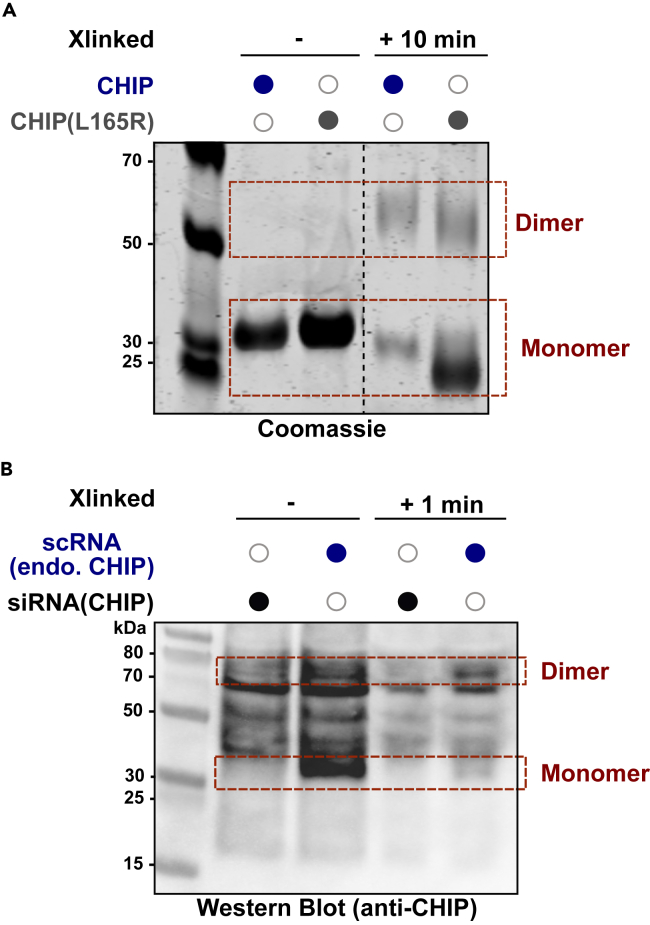
Dynamic conformation of CHIP as determined from recombinant proteins and lysates of HEK293T cells upon cross-linking with glutaraldehyde (A) Coomassie gel showing an expected result of a cross-linking experiment with recombinant CHIP (wild-type) and the monomeric CHIP mutant CHIP(L165R). In the non cross-linked samples, the recombinant proteins are present in monomeric forms. After cross-linking for 10 min at 30°C, CHIP is predominantly present in dimeric and oligomeric forms, whereas the monomeric mutant CHIP(L165R) remains predominantly monomeric. Some CHIP(L165R) dimers can be observed due to concentration-dependent oligomerization. The dashed line shows manipulation of Coomassie gel, i.e., by cutting out samples that are not relevant to this study. (B) Western blot showing an expected result of a cross-linking experiment at a given time point. In the non cross-linked scRNA control samples, the most prominent species present is the monomeric form. After cross-linking for 1 min at 30°C, dimeric and oligomeric intensity levels increase while monomeric species decrease.

In the non cross-linked samples, the recombinant proteins are present in monomeric forms (Figure 1A). After cross-linking for 10 min at 30°C, CHIP is predominantly present in dimeric and oligomeric forms, whereas the monomeric mutant CHIP(L165R) remains predominantly monomeric. Low levels of CHIP(L165R) dimers can be detected due to concentration-dependent oligomerization. When using this assay with recombinant proteins, one can compare the band intensity (from the Coomassie-stained gel) of the wild-type form of the protein to an obligate monomer and/or dimer mutant. This is done to assess the running behavior of the wild type and the mutants to be able to better assess the different structural forms present in the cell crude lysate.

The purpose of the siRNA is to knock down the endogenously encoded CHIP in HEK293T cells (Figure 1B). This will be reflected in decreased CHIP protein levels. The scRNA acts as a transfection control to ensure that the addition of exogenous RNA does not affect the results. To confirm that the protein band is indeed our protein of interest, we compare the decreased or missing protein band of CHIP in the siRNA condition to the endogenous protein band of CHIP in the scRNA condition.

In the non cross-linked scRNA control samples, the monomeric form is the most dominant species present. After cross-linking for 1 min at 30°C, dimeric and oligomeric species become more prominent while monomeric species decrease.

## Limitations

In this protocol, chemical cross-linking is used as a measure to detect protein-protein interactions. When two monomeric proteins interact during cross-linking, they are covalently crosslinked as a dimeric protein. By using non-reducing sample buffer, the covalent bonds remain intact during gel electrophoresis, allowing the separation of monomeric, dimeric, and oligomeric protein sizes in SDS-PAGE and Western blot analysis. Unbiased cross-linking of endogenous proteins is an elegant approach with minimal risk of artifacts that may arise from over-expression or *in vitro* systems. However, there are some issues that should be considered, which are addressed in the following.

This protocol is optimized for the detection of monomeric and dimeric forms of the protein CHIP, and the maximum cross-linking time must be adjusted for any other protein of interest. To achieve this, cross-linked samples should be collected at different time points and analyzed by SDS-PAGE and Western blotting. For example, a good starting cross-linking time point scheme would be: 1 min, 3 min, 5 min, and 10 min. Generally, an informative time point is one where dimeric protein structures can still be observed. If there is a lack of signal at later time points, higher order oligomeric structures may have formed that are unable to enter the SDS gel pockets. Therefore, the cross-linking time must be shortened.

Not only homodimers will be covalently linked, but possibly also heterodimers or larger heterocomplexes as your target protein interacts with other proteins in the cell lysate. Typically, these protein-protein interactions do not result in significant Western blot signals, and the dominant signals should be related to the conformational sizes of your target protein. However, cross reactions cannot be excluded. Therefore, we would like to emphasize that the Western blot alone is only partially informative. Before testing an endogenous protein in cell lysates, it is highly recommended to perform the cross-linking experiment with the recombinant counterpart of your protein of interest to determine the sizes and running behavior of dimeric and oligomeric species. Furthermore, to further confirm the running behavior of the monomer counterpart of the protein CHIP, we have generated a mutant that cannot self-assemble (CHIP(L165R)) due to a point mutation in its dimerization interphase.^1^

Ultimately, many proteins that can self-assemble into dimers and higher oligomers are usually present as monomeric proteins at comparatively low levels. Therefore, monomers of low abundant endogenous proteins may be beyond the scope of detection by Western blotting.

## Troubleshooting

### Problem 1

The storage of your protein of interest is not compatible with the suggested storage buffer (related to chemical cross-linking using recombinant proteins, Step 1).

### Potential solution

• Since the storage buffer of your protein is diluted with HEPES buffer prior to the cross-linking reaction, you can use alternative buffers. However, you should be aware that the effectiveness of glutaraldehyde can be affected by the pH and composition of the buffer used. In general, glutaraldehyde is stable in a wide range of buffer conditions at neutral pH (pH 7.0 to 8.0 is recommended), but there are some issues to consider.
• Buffers containing primary amines may promote undesirable interactions with glutaraldehyde because glutaraldehyde reacts with primary amines.
• Phosphate buffers at high pH (above 8.0) may hydrolyze glutaraldehyde, affecting its stability and activity.
• Buffers containing reducing agents may interfere with the enzymatic activity of glutaraldehyde by potentially reducing the reactive aldehyde groups of glutaraldehyde required for the cross-linking reaction.
• Buffers containing high concentrations of metal ions should be avoided as they may interfere with the catalytic activity of glutaraldehyde, directly affect cross-linking or promote precipitation of proteins or buffer components.

### Problem 2

The volume you require for 80 μg of cells exceeds the final volume of 20 μL used for the cross-linking (related to Step 4).

### Potential solution

• If you are using 2e^6^ cells per condition, your lysate should be concentrated enough to yield 80 μg in 10–15 μL (optimal final lysate concentration between 4 μg/μL and 8 μg/μL in a final volume of 20 μL). If the volume required for 80 μg already exceeds the final volume of 20 μL, you will need to adjust the protocol, e.g., by using more cells, less lysis buffer, more HEPES buffer & cross-linking and quenching reagents, etc. It is important that the final volume does not exceed 50 μL in order to load the entire amount into the wells of a 10-well gel. 10-well gels typically accommodate a maximum of 50 μL per well.

### Problem 3

No visible Western blot signal at later time points (related to Step 21b).

### Potential solution

• If you experience a lack of signal at later time points, higher order oligomeric structures may have formed and are unable to exit the SDS gel pockets. Running the SDS gel electrophoresis at a lower voltage (∼100 V) for the first 20 min and then increasing the voltage may also help to avoid this problem.
• If this does not resolve the issue, decrease the concentration of glutaraldehyde, decrease the cross-linking duration, or optimize the final protein concentration.
• You can adjust the cross-linking time points according to your protein of interest. For instance, you can test different time points: 1, 5, 10 min of cross-linking and observe which of these time points allow you to clearly observe the change in the level of the monomer and dimer compared to the non-cross-linked control samples. It is important to note that for CHIP, we observed an increased dimer to high order oligomer change beyond 10 min of cross-linking as this might be the case for other proteins.

### Problem 4

The quality of the Western blot is too low and the bands appear blurred (related to step 21b).

### Potential solution

• We have observed differences in the quality of the Western blot results depending on the type of SDS gel used (4–12% gradient gel vs. 10% Bis-Tris gel). In our experience, the 10% Bis-Tris gels gave the best results in terms of resolution and reproducibility.
• If the Western blot bands appear blurred, which can be caused by the use of crude lysate, you can alternatively perform wet blotting at 4 °C at 70 V for 100 min. You can use standard Towbin blotting buffer (25 mM Tris, 192 mM Glycine, 20% Methanol, 0.1% SDS).

### Problem 5

There is no Western blot signal at all (related to 21b).

### Potential solution

• If the Western blot signal derived from the charge-coupled camera device is below the detection limit, you can alternatively use X-ray detection with HRP-conjugated secondary antibodies. The exposure time depends on the protein of interest and the amount of protein present in the sample.

## Resource availability

### Lead contact

Further information and requests for resources and reagents should be directed to and will be fulfilled by the lead contact, Thorsten Hoppe (thorsten.hoppe@uni-koeln.de).

### Technical contact

Questions about the technical specifics of performing the protocol should be directed to and will be answered by the technical contact, Leonie Müller (lmuel111@uni-koeln.de).

### Materials availability

No new unique reagents were generated in this study.

### Data and code availability

The protocol includes all data sets generated during this study.
